# Association of Serum Uric Acid with Metabolic Syndrome and Its Components: A Mendelian Randomization Analysis

**DOI:** 10.1155/2020/6238693

**Published:** 2020-02-22

**Authors:** Lu Wang, Tao Zhang, Yafei Liu, Fang Tang, Fuzhong Xue

**Affiliations:** ^1^Healthcare Big Data Institute of Shandong University, Jinan 250000, China; ^2^Department of Epidemiology and Health Statistics, School of Public Health, Shandong University, Jinan 250000, China; ^3^Center for Data Science in Health and Medicine, Shandong Provincial Qianfoshan Hospital, The First Affiliated Hospital of Shandong First Medical University, Jingshi Road 16766, Jinan 250014, China; ^4^Shandong Provincial Qianfoshan Hospital Affiliated to Shandong University, Jinan 250000, China

## Abstract

**Background:**

The role of uric acid on metabolic syndrome (MetS) has always been controversial. This study aims to explore associations between uric acid with MetS and its components in Chinese female health check-up population.

**Methods:**

1381 subjects constituted the longitudinal health check-up cohort. Health examination and genotyping were performed. Unadjusted and adjusted observational analyses were implemented to evaluate observational associations between uric acid with MetS and its components. Mendelian randomization analysis was performed to estimate the causal effect using variation at rs11722228 (*SLC2A9*) as an instrument for uric acid.

**Results:**

An increase of 65% in risk of MetS per standard deviation increase in uric acid was found using unadjusted observational analyses. This association attenuated on adjustment for potential confounders. Similar patterns were found in the association analyses of uric acid with hyperglycemia, hypertension, and dyslipidemia. Neither by performing unadjusted nor adjusted analysis did we see evidence for association of uric acid on overweight and obesity. Mendelian randomization analyses showed no evidence of causal association between uric acid and MetS and MetS components.

**Conclusions:**

We found no causal evidence to support that increased serum uric acid is a causal risk factor for MetS or its components. Hence, there remains no strong evidence for the effeteness of undergoing urate-lowering therapy to prevent the onset of MetS or cardiovascular disease in health management.

## 1. Background

Metabolic syndrome (MetS) is a constellation of risk factors for cardiovascular disease (CVD). Epidemiological studies have found that uric acid was an independent predictor of CVD [1] and was associated with elevated risk of MetS [2]. Urate-lowering therapy has been taken into consideration to improve cardiovascular outcomes [3]. However, Mendelian randomization approaches have proved that there is no strong evidence for causality of uric acid on ischaemic heart disease [4], which means no direct causal effect of uric acid on CVD. Yet, the causal link between uric acid and MetS may lead to an indirect effect on CVD. To discuss the rationality for high cardiovascular risk population to reduce blood uric acid level as a treatment remains to be discussed. The causality of uric acid on both MetS and its components needs to be investigated.

Cohort study design is a prevailing study design for epidemiologists to discover causal relationships between risk factors and outcome diseases. However, as a kind of observational studies, the cohort study also suffers from the drawbacks of etiological mechanism distort caused by the existence of confounding, bias, and reverse causation. To avoid such disadvantages, the Mendelian randomization method is designed to introduce a randomization scheme into an observational study [5]. According to Mendel's second law, genetic associations are less likely to be affected by confounding and have been increasingly used in causal inference [6–8]. The heritability of serum uric acid concentrations is estimated at 40–70% [9–11]. Genome-wide association studies (GWAS) have identified several genetic variants associated with uric acid [12]. Among them, *SLC2A9* (solute carrier family 2, member 9) in chromosomal region 4p16.1 is found to be strongly associated with serum uric acid [13] and has been employed as an instrument for causation detection in several Mendelian randomization research studies [4, 14].

The causality between uric acid and MetS and its components should provide theoretical support for effeteness of urate-lowering therapy on preventing the onset of MetS and its components and further lowering the risk of CVD. Therefore, the purpose of this study is to investigate the causal relationship between uric acid with both MetS and its components using a Mendelian randomization analysis.

## 2. Methods

### 2.1. Study Population

A prospective cohort study was generated from the Shandong multicenter longitudinal cohort for health management. This database provided questionnaires, physical examinations, and laboratory index information of participants who conducted the annual health examination at the centers. To control for gender bias, the study subjects were restricted in female population. Participants aged more than 20 years and had at least 2 health check-up records at Shandong Qianfo Mountain Hospital from 2010 to 2015 were included in the baseline of the prospective cohort study. Among them, genotyping analysis was performed on blood samples from 1404 randomly selected subjects. Participants with MetS, cardiovascular, or cerebrovascular diseases at their first check-up were excluded. Finally, a total number of 1381 females constitute the Chinese MetS cohort study. We further established four MetS component subcohorts from the original MetS cohort study by excluding subjects with specific MetS component at their baseline health check-up, respectively. The study protocol was approved by Shandong University, and informed consent was obtained from all study participants.

### 2.2. Data Collection

A standardized questionnaire data was collected by trained interviewers via face-to-face interviews. Information on socio-demographic factors, health and medication status, and lifestyle was included in the questionnaires. The general health examination was performed at the same time. Body mass index (BMI) was calculated as weight in kilograms divided by square of height in meter. All subjects were examined in the morning after overnight fasting. For each individual, fifteen milliliters of fasting blood was drawn and distributed into three tubes: two ethylenediaminetetraacetic acid anticoagulant tubes for plasma and DNA and one coagulation tube for serum. Uric acid, triglyceride (TG), total cholesterol (TC), low-density lipoprotein cholesterol (LDL-c), high-density lipoprotein cholesterol (HDL-c), and blood glucose levels were measured by the hospital's laboratory.

### 2.3. Metabolic Syndrome and Its Components

In this study, outcome information was collected and verified by combining laboratory indexes and self-reported disease history. MetS was defined according to the criteria recommended by the Chinese Diabetes Society in 2004. Patients with three or more of the following disturbances were diagnosed with metabolic syndrome, they are as follows: (1) overweight/obesity: BMI ≥25.0 kg/m^2^; (2) hyperglycemia: fasting blood glucose (FPG) ≥6.1 mmol/L and/or postload glucose (2hPG) ≥7.8 mmol/L, or previously diagnosed as type 2 diabetes mellitus (DM) and received treatment; (3) hypertension: systolic blood pressure (SBP)/diastolic blood pressure (DBP) ≥140/90 mmHg, or previously diagnosed as hypertension and received treatment; and (4) dyslipidemia: TG level ≥1.7 mmol/L, and/or HDL-C level <0.9 mmol/L (men) or <1.0 mmol/L (women) [15].

### 2.4. Serum Uric Acid

Serum uric acid was measured by laboratory examination at baseline health check-up. Serum uric acid was internally standardized by 5-year age groups using a *z* score ([Supplementary-material supplementary-material-1]) over the Chinese cohort. Results were therefore relative risks per standard deviation increase in uric acid.

### 2.5. Genotyping

In this study, rs11722228, a functional mutation locus of urate transporter gene *SLC2A9*, was selected as an instrumental variable for the Mendelian randomized design [4, 16]. The design and synthesis of primers, DNA extraction, and genotyping were completed using peripheral blood samples. According to SNP sequence information, primer design software Assay design3.1 (Sequenom) was performed to design PCR and single-base extension primer. DNA from the blood sample was extracted by using the finished kit, and OD value detection was performed by NanoDrop2000, 1.25% agarose gel electrophoresis, and DNA quality control, and then, DNA was transferred to a 96-well plate at −20°C storage; the genotyping of rs11722228 was conducted by the Sequenom Mass Array system. Genotypes of *SLC2A9* (rs11722228) were coded by applying an additive genetic model based on information from a genome-wide association study [16].

### 2.6. Other Covariables

In this study, data were available for age, smoking status, and alcohol status from questionnaire information. According to the respondents' self-reported smoking status, smoking was categorized as smokers (current smokers or ex-smokers) and nonsmokers. Alcohol status was categorized as self-reported drinkers (current drinkers or ex-drinkers) and nondrinkers. Other variables were dichotomized as ‘yes or no' on the basis of the responses to questions on the use of lipid or blood pressure or blood glucose lowering drugs. Observational associations were estimated with and without adjustment for potential confounding factors.

### 2.7. Statistical Analysis

For *SLC2A9* (rs11722228), we investigated deviation from the Hardy–Weinberg equilibrium using a Pearson *χ*2 test. Cox proportional hazards regression models were performed to estimate the associations of the SNP and uric acid and other covariates with MetS and its components. General linear models were applied to examine the relationships of the genotype and uric acid. We tested the associations of the individual SNP with category covariates by logistic regression models.

**Figure 1:**
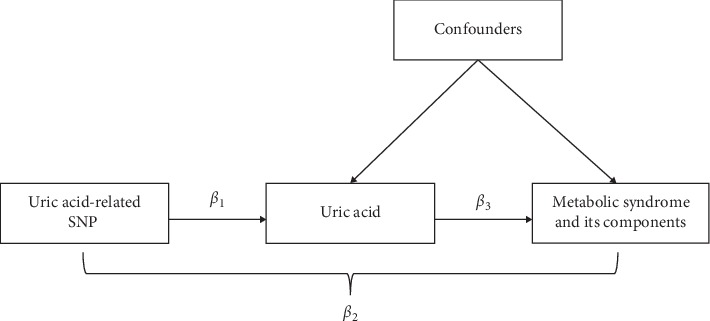
Schematic representation of the Mendelian randomization analysis.

In this study, we performed both observational analysis, which was conducted using the cox proportional hazards regression model to test association between uric acid and outcomes with and without confounding and Mendelian randomization analysis. Mendelian randomization analysis was conducted to estimate causal effects. A schematic presentation of the Mendelian randomization analysis is shown in Figure 1. In a Mendelian randomization design, the causal effects of uric acid with MetS and its components could be estimated as *β*_3_ estimates. The *β*_3_ estimates for the SNP to evaluate the association between uric acid and risk of MetS, and its components can be calculated from the direct measurements *β*_1_ (the estimate of effect size of the SNP on uric acid) and *β*_2_ (the estimate of effect size of the SNP on MetS and its components) as *β*_3_ = *β*_2_/*β*_1_ [17]. The SE of *β*_3_ is calculated using the delta method. That is, $S_{3}=\sqrt{1/\beta_{1}^{2}S_{2}^{-2}}$, where *S*_2_ is the SE of *β*_2_ [18]. In this study, *β*_1_ is the estimate of effect size of the SNP on standardized uric acid and *β*_2_ is the log_*e*_ HR estimate of MetS and its components for the SNP. The HR for MetS and its components associated with each 1 SD lower genetically determined uric acid can be given by exp (overall *β*_3_). All analyses were performed using R version 3.4.2.

## 3. Results

From 2010 to 2015, 1381 eligible participants aged from 21 to 81 years were enrolled in the Chinese (CH) MetS cohort. The median follow-up years were 2.01. At the end of the follow-up period, 61 subjects developed MetS. The incidence density was 19.96/1000 person-years. Detailed description of MetS components cohorts is in [Supplementary-material supplementary-material-1].

Table 1 shows baseline characteristics for CH MetS and four MetS component cohorts. The baseline mean age was 39.48 for entire cohort subjects and was 53.98 for individuals who developed MetS, 42.90 for individuals who developed overweight and obesity, 51.46 for individuals who developed hyperglycemia, 45.08 for individuals who developed hypertension, and 45.90 for subjects who developed dyslipidemia, respectively. The mean level of serum uric acid was 257.33 *μ*mol/L (standard deviation (SD): 52.80 *μ*mol/L) for the whole population and 286.26 *μ*mol/L (SD: 62.94 *μ*mol/L), 258.56 *μ*mol/L (SD: 50.37 *μ*mol/L), 273.63 *μ*mol/L (SD: 57.91 *μ*mol/L), 269.64 *μ*mol/L (SD: 54.22 *μ*mol/L), and 263.98 *μ*mol/L (SD: 52.24 *μ*mol/L) for individuals developed MetS or four MetS components, respectively. Serum uric acid level was significantly associated with all four outcomes except for overweight and obesity in univariate cox proportional hazards regression analyses. The *P* value for serum uric acid level associated with overweight and obesity was 0.088. For the prevalence of smoking and drinking was low in Chinese female population, the corresponding associations with health outcomes were not significant in most cases. Therefore, we did not further adjust those two confounders in the following analyses.

There was no evidence for genotype of rs11722228 that deviated from Hardy–Weinberg equilibrium. The association between variation at *SLC2A9* (rs11722228) and uric acid was roughly linear ([Supplementary-material supplementary-material-1]). Mean levels of uric acid showed an increase in standard deviation of 0.23 (95% CI: 0.14 to 0.31) for each additional T allele in CH MetS cohort. The genotype of rs11722228 was not associated with MetS and its components. The hazard ratios and its 95% confidence interval (CI) are given in Table 2. *SLC2A9* (rs11722228) was not associated with potential confounders: age, smoking, and drinking status ([Supplementary-material supplementary-material-1]).

**Figure 2:**
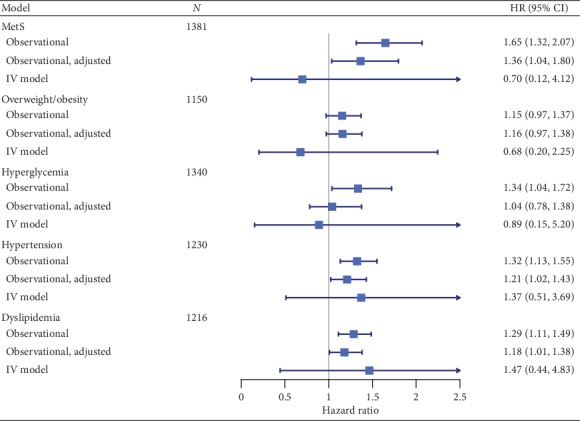
Forest plot showing observational and instrumental variable estimates of the effect of standardized serum uric acid on MetS and its components in the Chinese cohort study. Observational: cox proportional hazards regression model without adjusting for confounding (age, baseline MetS components, and drug information); observational, adjust: cox proportional hazards regression model adjusting for confounding (age, baseline MetS components, and drug information); IV model: Mendelian randomization model.

Based on observational estimates, an increase in uric acid of one standard deviation was associated with hazard ratios for MetS of 1.65 (95% CI: 1.32 to 2.07) in the CH cohort (Figure 2). After adjusting for age, baseline MetS components, and drug information, the hazard ratio remained significant (HR: 1.36, 95% CI: 1.04 to 1.80).

Similar patterns were also found in association with hypertension and dyslipidemia. Before adjustment, the significant observational associations were observed between serum uric acid with hypertension and with dyslipidemia. After adjustment for corresponding confounders, the association remained significant with hazard ratio as 1.21 (95% CI: 1.02 to 1.43) for hypertension and 1.18 (95% CI: 1.01 to 1.38) for dyslipidemia, respectively.

The increase of serum uric level was also associated with increased risk of hyperglycemia with hazard ratio corresponding to 1.34 (95% CI: 1.04 to 1.72). Nevertheless, this association was not observed after adjustment for confounders (HR: 1.04, 95% CI: 0.78 to 1.38). Before adjustment or not, there was no evidence for association between uric acid and overweight and obesity.

To explore the causal relationships, we further performed Mendelian randomization analysis to estimate the causal effect size of genetically determined uric acid on MetS and its components in CH MetS cohort. The causal hazard ratios for MetS was 0.70 (95% CI: 0.12 to 4.12) per standard deviation increase in uric acid. For four MetS components, there were no evidence for causal associations too (Figure 2). The causal estimates were 0.68 (95% CI: 0.20 to 2.25), 0.89 (95% CI: 0.15 to 5.20), 1.37 (95% CI: 0.51 to 3.69), and 1.47 (95% CI: 0.44 to 4.83) for overweight and obesity, hyperglycemia, hypertension, and dyslipidemia, respectively.

## 4. Discussion

We found no evidence for the casual effect of uric acid on the risk of MetS or MetS components. An increase of 65% in risk of MetS per standard deviation increase in uric acid was found using unadjusted observational analyses. This association attenuated on adjustment for potential confounders. The similar pattern was present in the association analyses of uric acid on hyperglycemia, hypertension, and dyslipidemia. Neither by performing unadjusted nor adjusted analyses did we see evidence for association of uric acid on overweight and obesity. Mendelian randomization analyses showed no evidence of causal association between uric acid and MetS or MetS components.

Uric acid is a metabolite derived from the purine catabolism [19]. Although some observational research studies have seen uric acid as an independent risk factor of MetS [20–23], experimental study has proposed uric acid as a powerful antioxidant and being able to protect against cardiovascular disease and some cancers [24]. In this study, to avoid confounding and reverse causation, Mendelian randomization analysis was performed to estimate the causal effect of uric acid on MetS and its components.

The role of uric acid in the development of MetS has always been controversial. In this study, unadjusted and adjusted observational analyses showed positive relationship between increased serum uric acid level and increased risk of MetS and four MetS components. Mendelian randomization analysis, though not significant, indicated uric acid as a protective factor of MetS, overweight and obesity, and hyperglycemia. Those findings which were consistent with experimental studies suggested uric acid as an antioxidant [25, 26]. This result also consistent with a previous study found, although not significant, a negative causal effect on MetS [27]. A recent longitudinal cohort study in Korea also detected protective effects of uric acid on MetS [28]. One possible explanation would be that the elevated uric acid level is a positive response to the diseases associated with oxidative stress process [29].

Estimates derived from this Mendelian randomization here resumptively agree with certain recent studies. McKeigue et al. implemented Bayesian methods for instrumental variable analysis and failed to find evidence for a casual effect of uric acid on MetS in a Scottish population isolate [27]. Use of the Mendelian randomization approach found no evidence for a causal link between uric acid and type 2 diabetes [14]. Furthermore, using the genetic score for hyperuricaemia in a Mendelian randomization approach also did not provide evidence of causal effects on cardiovascular disease risk factors including gout, blood pressure, glucose, chronic kidney disease, and coronary heart disease [30]. Evidence from another Mendelian randomization approach also did not support a causal role of uric acid on type 2 diabetes, coronary heart disease, ischaemic stroke, and heart failure [31]. On the contrary, an Mendelian randomization study employed rs16890979 (*SLC2A9*) as the instrument and found causal associations between uric acid with hypertension [32]. Those indicate that further research studies should focus on the complex genes and uric acid function.

The major strength of our study includes the combination of observational analyses and causal relationship analyses in a prospective cohort study. In this study, the Mendelian randomization method was performed to control confounding and reverse causation, and therefore, the causal effect of uric acid on MetS and its components was derived. In addition, the results derived from Mendelian randomization suggested that there remained no strong evidence for clinical practice of urate-lowering therapy as MetS preventing treatment.

The limitation of this research is as follows: first, the definition of MetS components may be influenced by measurement bias; second, being limited by the study follow-up period, the sample size of CH cohorts was not quite large; and last, using single SNP as an instrument may be limited by pleiotropy and complicated gene interactions. Hence, further studies need to explore the pleiotropy and interactions between uric acid-related genes, and this may help finding explanations for observational associations between uric acid and health outcomes.

## 5. Conclusions

In conclusion, we found no causal evidence to support that serum uric acid is a causal factor of metabolic syndrome and its components. Hence, there remains no strong evidence for the effeteness of undergoing urate-lowering therapy to prevent the onset of MetS or cardiovascular disease in health management.

## Figures and Tables

**Table 1:** Baseline characteristics of CH MetS cohort and those who developed MetS, overweight and obesity, hyperglycemia, hypertension, and dyslipidemia during follow-up.

Characteristic	Entire cohort (*n* = 1381)	MetS (*n* = 61)	Overweight and obesity (*n* = 125)	Hyperglycemia (*n* = 54)	Hypertension (*n* = 144)	Dyslipidemia (*n* = 169)
Age, year	39.48 (12.05)	53.98 (12.03)^*∗∗*^	42.90 (11.27)^*∗*^	51.46 (12.90)^*∗∗*^	45.08 (11.90)^*∗∗*^	45.90 (12.50)^*∗∗*^
Smoker, *n* (%)	5 (0.36)	0 (0.00)	2 (1.60)^*∗*^	0 (0.00)	1 (0.69)	0 (0)
Drinker, *n* (%)	89 (6.46)	4 (6.67)	11 (8.87)	5 (9.43)	10 (6.94)	9 (5.36)
BMI (kg/m^2^)	22.28 (3.07)	26.59 (3.14)^*∗∗*^	23.61 (1.36)^*∗∗*^	25.43 (3.26)^*∗∗*^	24.21 (3.60)^*∗∗*^	23.70 (3.04)^*∗∗*^
FBS (mmol/L)	5.02 (0.53)	5.48 (0.81)^*∗∗*^	5.12 (0.63)^*∗*^	5.53 (0.35)^*∗∗*^	5.21 (0.66)^*∗∗*^	5.18 (0.60)^*∗∗*^
SBP (mmHg)	119.38 (15.04)	134.18 (15.90)^*∗∗*^	121.34 (15.13)^*∗*^	130.80 (16.08)^*∗∗*^	126.56 (9.08)^*∗∗*^	125.41 (15.64)^*∗∗*^
DBP (mmHg)	72.79 (9.77)	79.67 (9.92)^*∗∗*^	73.34 (9.18)^*∗*^	77.39 (12.35)^*∗∗*^	78.22 (7.32)^*∗∗*^	75.70 (9.90)^*∗∗*^
TG (mmol/L)	0.95 (0.53)	1.53 (0.85)^*∗∗*^	1.07 (0.46)^*∗*^	1.40 (0.93)^*∗∗*^	1.17 (0.69)^*∗∗*^	1.12 (0.33)^*∗∗*^
LDL-c (mmol/L)	2.65 (0.68)	3.13 (0.66)^*∗∗*^	2.71 (0.61)	3.01 (0.64)^*∗∗*^	2.89 (0.69)^*∗∗*^	2.97 (0.63)^*∗∗*^
HDL-c (mmol/L)	1.59 (0.30)	1.43 (0.29)^*∗∗*^	1.55 (0.26)^*∗*^	1.49 (0.35)^*∗*^	1.57 (0.31)^*∗*^	1.52 (0.24)^*∗∗*^
Hypoglycemia drugs, *n* (%)	14 (1.02)	4 (7.02)^*∗∗*^	5 (4.03)^*∗*^	0 (0)	6 (4.20)^*∗∗*^	2 (1.21)
Antihypertensive drugs, *n* (%)	46 (3.41)	11 (20.00)^*∗∗*^	4 (3.36)	5 (10.00)^*∗*^	0 (0)	12 (7.55)^*∗*^
Lipid lowering drugs, *n* (%)	15 (1.12)	2 (3.77)	2 (1.64)	1 (2.00)	2 (1.46)	0 (0)
UA (*μ*mol/L)	257.33 (52.80)	286.26 (62.94)^*∗∗*^	258.56 (50.37)	273.63 (57.91)^*∗*^	269.64 (54.22)^*∗∗*^	263.98 (52.24)^*∗*^

Data are mean (standard deviation) for continuous variables and number (%) of nonmissing observations for each binary variable. BMI = body mass index; FBS = fasting blood glucose; SBP = systolic blood pressure; DBP = diastolic blood pressure; TG = triglycerides; HDL-C = high-density lipoprotein cholesterol; UA = uric acid; MetS = metabolic syndrome. ^*∗*^*P* < 0.05, univariate cox proportional hazards regression analyses with MetS or four MetS components. ^*∗∗*^*P* < 0.001, univariate cox proportional hazards regression analyses with MetS or four MetS components.

**Table 2:** Association of *SLC2A9* (rs11722228) associated with exposures and outcomes.

	Coefficient/HR	95% confidence interval	*P* value
Exposure			
Standardized serum uric acid	0.23	(0.14, 0.31)	<0.001
Outcomes			
MetS	0.92	(0.62, 1.38)	0.692
Overweight & obesity	0.91	(0.69, 1.21)	0.524
Hyperglycemia	0.97	(0.64, 1.47)	0.896
Hypertension	1.08	(0.84, 1.39)	0.531
Dyslipidemia	1.08	(0.85, 1.36)	0.53

Data are coefficient (95% CI) for association with standardized serum uric acid and hazard ratio (95% CI) for association with outcomes; genotypes under an additive model.

## Data Availability

The data used to support the findings of this study are available from the corresponding author upon request.

## Abbreviations

MetS: Metabolic syndrome
CVD: cardiovascular disease
BMI: Body mass index
CH: Chinese
TG: Triglyceride
TC: Total cholesterol
LDL-c: Low-density lipoprotein cholesterol
HDL-c: High-density lipoprotein cholesterol
FPG: Fasting blood glucose
2hPG: Postload glucose
DM: Type 2 diabetes mellitus
SBP: Systolic blood pressure
DBP: Diastolic blood pressure.

## Supplementary Materials

Supplementary Materials Supplementary Table S1: means and standard deviations of uric acid (mmol/L) by age groups used to generate standardized UA (*Z*-scores) in the CH cohort. Supplementary Table S2: basic description of CH MetS cohort and its four component cohorts. Supplementary Figure S1: mean serum uric acid by SLC2A9 (rs11722228) genotype in the Chinese (CH) cohort study (means in points and 95% confidence intervals in bars). Supplementary Figure S2: forest plot showing estimates of genetic risk scores on potential confounders in Chinese cohort.
