# Supplementary material for: Association of Serum Uric Acid with Metabolic Syndrome and Its Components: A Mendelian Randomization Analysis
Source: Biomed Res Int. 2020 Feb 22;2020:6238693. doi: 10.1155/2020/6238693 (PMC7063870; doi:10.1155/2020/6238693)
Supplement: Supplementary Materials — Supplementary Table S1: means and standard deviations of uric acid (mmol/L) by age groups used to generate standardized UA (Z-scores) in the CH cohort. Supplementary Table S2: basic description of CH MetS cohort and its four component cohorts. Supplementary Figure S1: mean serum uric acid by SLC2A9 (rs11722228) genotype in the Chinese (CH) cohort study (means in points and 95% confidence intervals in bars). Supplementary Figure S2: forest plot showing estimates of genetic risk scores on potential confounders in Chinese cohort. [file 6238693.f1.zip › 6238693.f1/Supply_Tables.docx]

**Table S1.** Means and standard deviations of uric acid (*μmol*/L) by age group used to generate standardized UA (Z-scores) in CH cohort.

| Age group | N | Serum uric acid |
| --- | --- | --- |
| 21-24 | 71 | 268.2 (55.7) |
| 25-29 | 220 | 263.4 (53.9) |
| 30-34 | 313 | 254.1 (49.2) |
| 35-39 | 207 | 247.6 (49.8) |
| 40-44 | 151 | 245.8 (48.9) |
| 45-49 | 147 | 249.5 (49.8) |
| 50-54 | 91 | 271.2 (57.6) |
| 55-59 | 82 | 268.1 (52.6) |
| 60-69 | 63 | 271.9 (57.2) |
| 70-81 | 36 | 278.2 (67.1) |

**Table S2.** Basic description of CH MetS cohort and its four components cohorts

| Cohort | *N* | Incidence cases (/1,000 person-year) | Median follow-up years (SD) |
| --- | --- | --- | --- |
| MetS | 1381 | 61 (19.96) | 2.01 (1.15) |
| Overweight/Obesity | 1150 | 125 (51.12) | 2.01 (1.13) |
| Hyperglycemia | 1340 | 54 (18.26) | 2.01 (1.16) |
| Hypertension | 1230 | 144 (55.29) | 2.01 (1.13) |
| Dyslipidemia | 1216 | 169 (65.35) | 2.01 (1.15) |
