# Supplementary figures and images for: Association of Serum Uric Acid with Metabolic Syndrome and Its Components: A Mendelian Randomization Analysis
Source: Biomed Res Int. 2020 Feb 22;2020:6238693. doi: 10.1155/2020/6238693 (PMC7063870; doi:10.1155/2020/6238693)

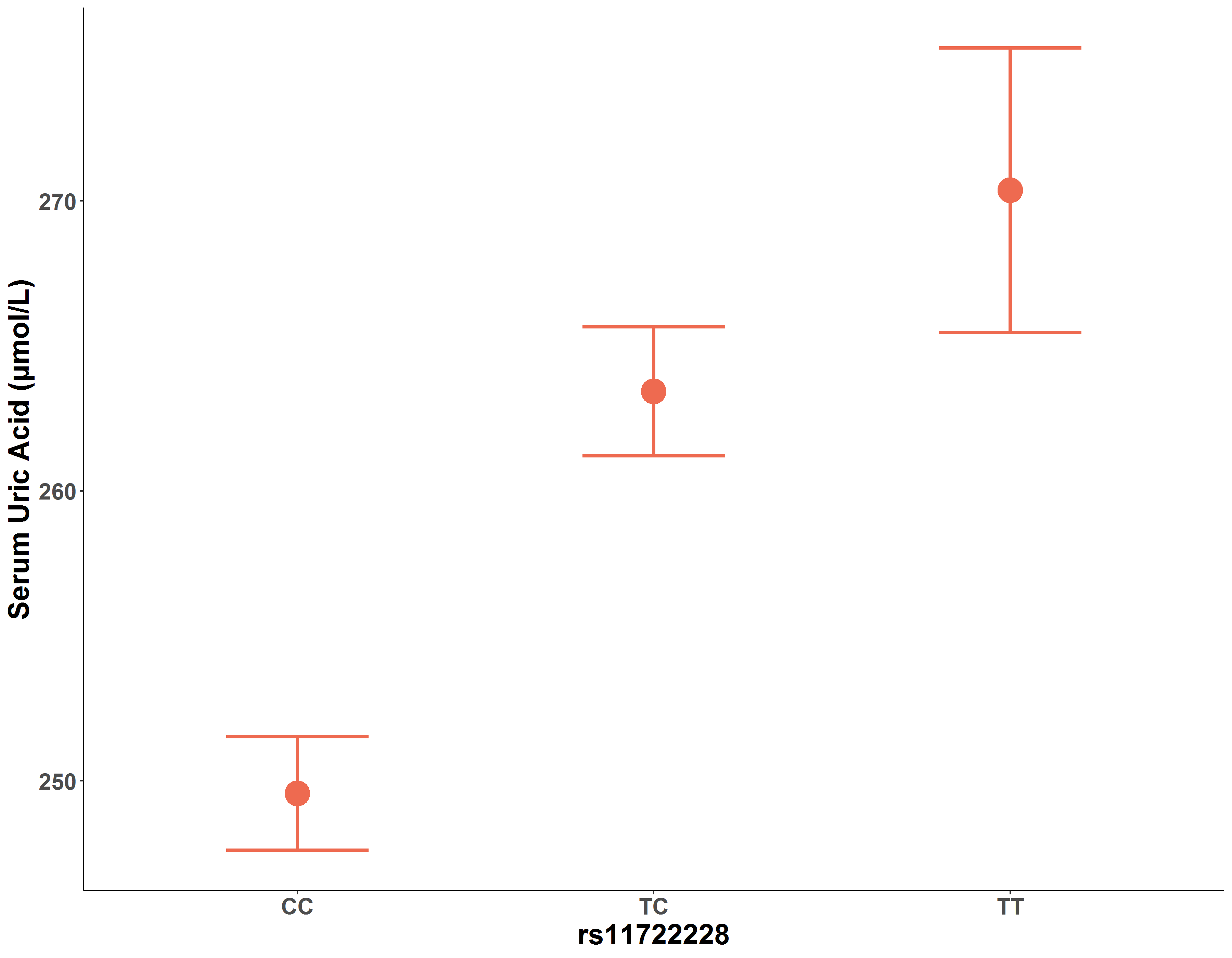

Supplement: Supplementary Materials — Supplementary Table S1: means and standard deviations of uric acid (mmol/L) by age groups used to generate standardized UA (Z-scores) in the CH cohort. Supplementary Table S2: basic description of CH MetS cohort and its four component cohorts. Supplementary Figure S1: mean serum uric acid by SLC2A9 (rs11722228) genotype in the Chinese (CH) cohort study (means in points and 95% confidence intervals in bars). Supplementary Figure S2: forest plot showing estimates of genetic risk scores on potential confounders in Chinese cohort. [file 6238693.f1.zip › 6238693.f1/Fig_S1.tiff]

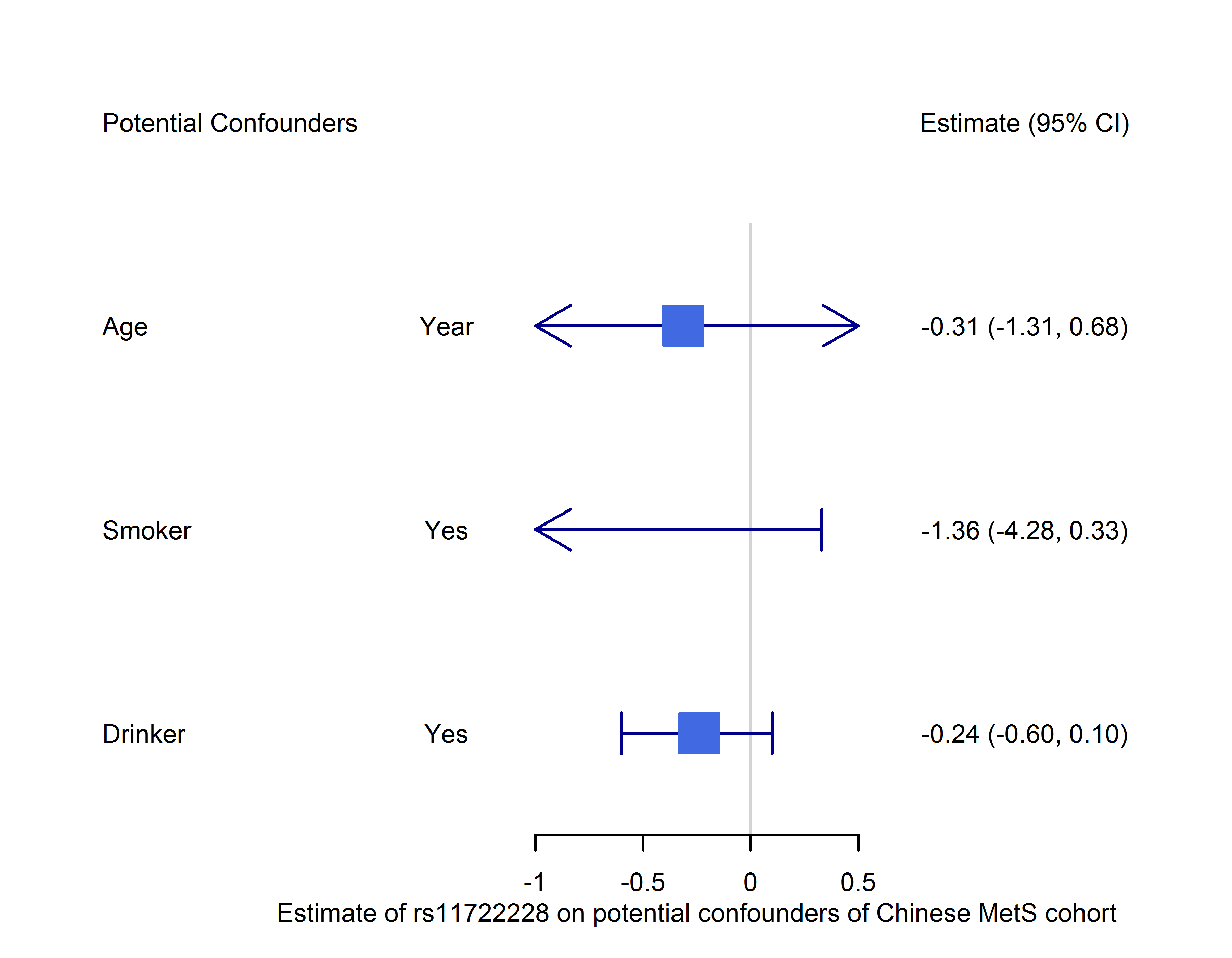

Supplement: Supplementary Materials — Supplementary Table S1: means and standard deviations of uric acid (mmol/L) by age groups used to generate standardized UA (Z-scores) in the CH cohort. Supplementary Table S2: basic description of CH MetS cohort and its four component cohorts. Supplementary Figure S1: mean serum uric acid by SLC2A9 (rs11722228) genotype in the Chinese (CH) cohort study (means in points and 95% confidence intervals in bars). Supplementary Figure S2: forest plot showing estimates of genetic risk scores on potential confounders in Chinese cohort. [file 6238693.f1.zip › 6238693.f1/Fig_S2.tiff]
